# Proteomics investigation of human sera for determination of postoperative indicators of pulmonary cystic echinococcosis

**DOI:** 10.1186/s13019-023-02109-4

**Published:** 2023-01-11

**Authors:** Fatemeh Sadat Sadjjadi, Homa Hajjaran, Bahareh Sedaghat, Parviz Mardani, Seyed Mahmoud Sadjjadi

**Affiliations:** 1 Proteomics Research Center, Faculty of Paramedical Sciences, Shahid Beheshti University of Medical Sciences, Tehran, Iran; 2 Department of Medical Parasitology and Mycology, School of Public Health, Tehran University of Medical Sciences, Tehran, Iran; 3 Department of Parasitology and Mycology, School of Medicine, Shiraz University of Medical Sciences, Shiraz, Iran; 4 Department of Surgery, School of Medicine, Shiraz University of Medical Sciences, Shiraz, Iran

**Keywords:** Proteomics, Lungs cystic echinococcosis, Follow-up, 2‐DE, LC–MS/MS, Human serum

## Abstract

**Background:**

Cystic echinococcosis (CE)/hydatidosis is an important zoonotic parasitic disease caused by the larval stage of *Echinococcus granulosus*. The disease is a major health problem all over the world. Finding specific and sensitive biomarkers for follow-up of CE in patients after surgery is essential. Using proteomics methods, the present study aimed to evaluate post-surgical treatment by finding probable biomarker/s in the serum of human lungs CE.

**Methods:**

A total of 24 human sera were tested. These sera included eight confirmed lung/s CE patients sera before surgery (BS), eight sera 12 months post-surgery (12MPS) as well as eight control sera from healthy people. Proteomics methods including 2DE and LC–MS/MS were performed on the specimens followed by bioinformatics analysis. Differentially expressed proteins (DEP) were detected and, separately integrated with protein–protein interaction (PPI) data to construct the PPI network.

**Results:**

A total of 171 protein spots were detected in three groups including BS, 12MPS, and control groups; of which a total of 106 DEP have been expressed based on fold changes > = 2 and *p*-value < 0.05. More analysis was performed and a total of 10 protein spots were selected for identification by mass spectrometry showing the following proteins: APOA1, BGN, SPP2, EAF1, ACOXL, MRPL55, MCTP2, SEPTIN1, B4GALNT1, and ZNF843. Based on centrality parameters of the PPI network (degree and betweenness) five Hub-bottlenecks proteins with significant centrality values were found including APOA1, BGN, SPP2, EAF1, and ACOXL.

**Conclusion:**

This study showed five proteins as hub-bottleneck proteins; of which APOA1 was more prominent. It can be concluded that a change in expression of this protein in patients’ sera could be used as an indicator tool for the achievement of lungs CE surgical therapy.

## Introduction

Cystic echinococcosis(CE)/hydatidosis is an important zoonotic parasitic disease caused by the larval stage or metacestode of the tapeworm *Echinococcus granulosus* [1, 2]. The disease is still a major health problem all over the world including Iran [3–5]. Human cases of the disease are routinely reported in different hospitals of country [4–7]. The disease has been listed as one of the 17 neglected tropical diseases (NTDs) recognized by the World Health Organization [8]. Human can be infected via consuming the parasite eggs leading to cyst formation especially in the liver followed by lungs and other organs [2, 9].

Different species/genotypes of *Echinococcus* including *E. granulosus* sensu stricto (s.s) (G1–G3), *E. equinus* (G4), *E. ortleppi* (G5), *E. canadensis* (G6–G10), *E. multilocularis*, *E. vogeli*, *E. oligarthrus*, *E. felidis*, and *E. shiquicus* has been reported in the world [10–12]. A number of above species and genotypes including *E. granulosus* s.s, *E. canadensis, E. ortleppi* have been reported from different organs of humans and animals in Iran [5, 13–17]. Diagnosis and treatment of lungs CE is difficult due to confusion with other lung diseases and the fact that infection may remain asymptomatic or for a long time and then manifest itself as a severe and debilitating condition [9, 18, 19]. Radiological studies are the primary step in the detection and evaluation of pulmonary CE cysts. Considering the cost and availability, chest X-rays are still the most used procedure. In complicated CE cysts, CT is the imaging of choice. It can recognize the details of the lesions and their surrounding structures to help the differential diagnoses and uncover additional smaller cysts which are not usually detectable by conventional chest X-ray. The use of ultrasound in lung lesions is limited and is applicable in lesions close to the thoracic wall [20, 21]. Usually, the treatment of CE is performed through surgical procedure and, drug therapy. Due to the possibility of cyst rupture during surgery, the possibility of forming secondary cysts and recurrence of the disease, follow-up of patients with CE at regular intervals after surgery is essential [22]. Post-surgical follow up of CE is usually uses ultrasonography and serological methods [23, 24]. Ultrasonography which is usually used for post follow up of liver cyst surgery is not effective in lungs CE [2]. Currently, there are no markers for evaluation of surgical and drug therapy on CE patients [25]. Evaluation of antibody in sera of treated patients is not recommended due to persistent high level of antibody even after removal of cyst for several months [26].

Evaluation of antigens in sera although is more specific but possess low sensitivity [27]. So, it is essential to find new methods for monitoring and follow up of the treated human CE patients [19]. It seems that the proteomic identification could improve the understanding of biochemical and immunological characteristics of CE [28–30].

The present study using proteomics methods was aimed to find probable indicator/s biomarker/s in serum of lungs CE patients before surgery and twelve months post-surgery as evaluation criteria for achievement of lungs surgical therapy.

## Materials and methods

### Sample collection

A total of 24 human sera from different counties of Fars province, southern Iran were collected and stored at – 20 °C until use. In this region *E. granulosus* sensu stricto followed by *E. canadensis* (G6/7) are the most frequent species/genotypes in lungs CE [5]. The pulmonary cyst in the patients were classified according to the location of the cysts and their size. The rate of cysts in the left lobe were more than right lobe. The cysts’ size was classified as small (0–4 cm), medium (5–9 cm), and large (10–15 cm). About 50% of the cysts were small size followed by medium and large size.

The samples were transferred to the Helminthology Research Laboratory at the School of Medicine, SUMS, Iran, for further actions. These sera were included eight confirmed lung/s CE patients sera before surgery (BS), eight sera 12 months post-surgery (12MPS) as well as eight control sera from healthy people. The healthy samples were selected from people who had no sign and symptoms of CE and their sera was negative by ELISA test. After surgery a number of cases had protoscoleces in their CE cyst which was recognizable by microscopy. BS and 12MPS sera samples were collected from the same patients. Gender distribution of the patients showed the male (62%) and female (38%). Patients’ age ranged from under 10 to more than 70 years. All patients and control people had Iranian nationality. Informed consent was obtained from all individual participants included in the study.

### Protein separation by two dimensional gel electrophoresis (2-DE)

All sera in each group (BS, 12MPS and control) were thawed at room temperature, vortexed on ice, and pooled separately. Protein assay was performed on every pooled sera group using Bradford method [31]. Immobilized pH gradient (IPG) 7 cm strips (pH 3–10) (Bio-Rad, USA) were used for the first dimension of 2-DE. A diluted sample was made by adding 75 μl of each pooled sample in 50 μl of rehydration buffer (7 M urea, 2 M thiourea, 2% CHAPS, 50 mM DTT, 0.5% Bio-Lyte ampholyte, 0.001% bromophenol blue).

The diluted samples were loaded on IPG strips and incubated at room temperature for 16–18 h. The first dimension of 2-DE was performed with the following voltage and time schedule: (50 v, linear, 4 h), (250 v, linear, 1 h), (1000 v, linear, 1 h), (2000 v, linear, 1 h), (4000 v, linear, 2 h) and (4000 v, rapid, 10 h). The device turned off at 13000 v.

In the next step, the gels were equilibrated twice in equilibration buffers including: 6 M urea, 87% glycerol, 2% SDS, 0.375 M Tris, (pH = 8.8), 0.002% bromophenol blue, once by 1% DTT and once by 2.5% iodoacetamide, for 15 min.

To run the second dimension of 2DE, SDS PAGE was performed. IPG 7Cm strips (pH3-10) were transferred into SDS PAGE formed of 12% polyacryleamide gel and sealed with agarose. The gels transferred to electrophoresis tank with running buffer. Electric current was set at 100 v for 105 min. The 2-DE experiments were repeated three times for each group [30].

### Gel staining and gel de-staining

After completion of 2DE, the gels were removed and washed three times with deionized water for10 minutes for each step. KANG staining method [32] was performed for gels staining as follows: The gels were fixed in fixative solution (30 ml ethanol 30%, 2 ml phosphoric acid 2%, 6 ml DW) for 3 h. Then gels were washed 2 times with DW. Gels transfer in staining solution (0.4 gr Coomassie Brilliant Blue G-250, 100 gr aluminium solphate 5%, 200 ml ethanol 10% and 188 ml orthophosphoric acid 85%) for 21 h.

Gels were washed twice and transferred to the de-staining solution (200 ml ethanol 10%, 47 ml phosphoric acid 2%, DW) for 1 h while shaking.

### Statistical analysis

Statistical analysis was performed using Progenesis SameSpots software. A densitometer GS-800 (BioRad) was used for scanning image of 2DE gels. The images were analyzed using Progenesis SameSpots software to compare the processed gels for detecting the protein spots, evaluating their color intensity, spots alignment, and statistical analysis. One-way analysis of variance (ANOVA) and fold changes were used to determine the statistically significant changes in protein expression between groups. Spots with *p*-values < 0.05 and fold changes > = 2 are considered as significant spots.

### Mass spectrometry

A total of 10 significant spots which were selected according to their sharpness, density and the changes in protein expression between groups based on statistical analysis were incised and isolated followed by mass spectrometry for protein identification at Proteomics and Mass Spectrometry Core, Medical Plants and Drug Research Institute, University of Shahid Beheshti, Tehran, Iran. Matrix-assisted laser desorption/ionization mass spectrometry (Applied Biosystems 4800 MALDI- TOF/TOF) was used to analysis the samples. The collection solutions were desalted by passing through C18 Zip-Tip reverse phase chromatography pipette tip (Millipore, Bedford, USA) prior to MALDI- TOF analysis, according to the manufacturer’s instructions. The samples were spotted on MALDI plate mixed with an equal volume of matrix solution of sinapinic acid in 50% ACN containing 0.1% TFA, air dried, and analyzed with a MALDI-TOF/TOF mass spectrometer, operated in high linear positive mode. After that, the data were interpreted and processed using Data Explorer software version 4.0 (Applied Biosystems). The obtained mass diagram and data were analysed using Mascot database search (https://www.matrixscience.com/). Each identified protein was determined with UniProt accession number (https://www.uniprot.org/).

### Bioinformatics analysis

The UniProt accession number of differentially expressed proteins (DEPs) were subjected to STRING online database (https://string-db.org/) in order to construct the functional protein association network. The data obtained from STRING was subjected to Cytoscape v3.2.0 software. Protein–Protein Interaction (PPI) network was drawn for CE results obtained from HPRD, BIND, INTACT, MINT and STRING databases by the application Cytoscape v3.2.0 integrated to the whole network for all DEP in the experiments. Cytoscape extracted the centrality parameters including degree, bottleneck, and betweenness centrality, and analyze the network using special plugins as Cytohubba.

## Results

The 2DE method which was used for separating sera proteins in samples showed a total of 171 protein spots in three groups including BS, 12MPS and control. A total of 106 DEP have been expressed in three groups of sera tested, based on fold changes > = 2 and *p*-value < 0.05. A total of 51 out of 106 DEP spots have been increased their expression after surgery. Simultaneously, a total of 23 out of 106 DEP have been decreased their expression after surgery. In control group 32 DEP spots have been detected.

**Fig. 1:**
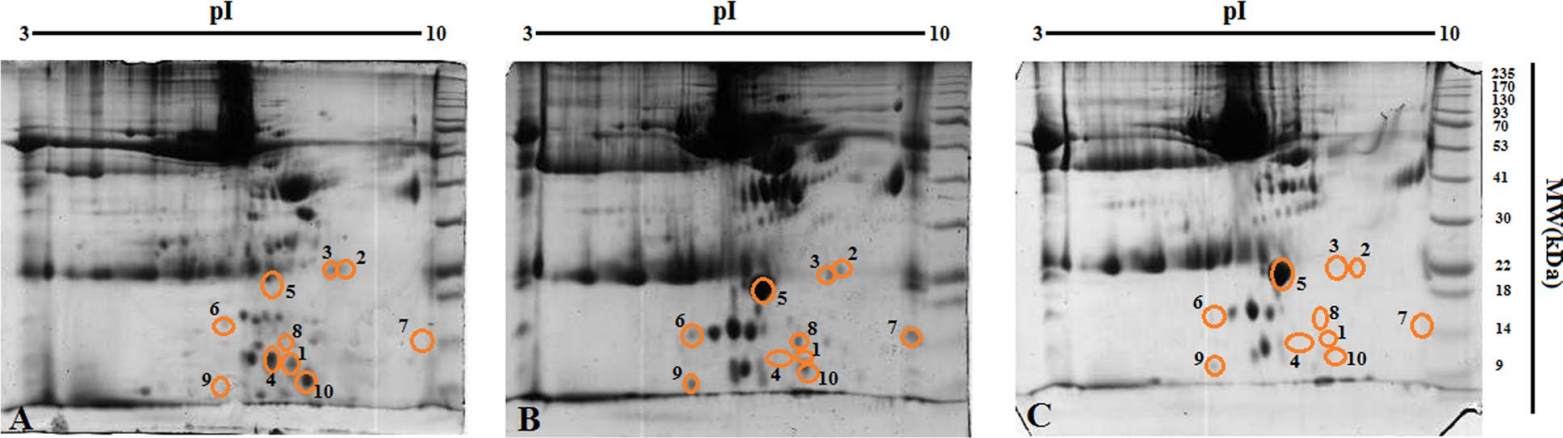
The 2DE gel scan image of CE patients’ sera. The identified protein spots indicated as circle. **A** control group, **B** before surgery group (BS) and **C** 12 Months post-surgery group (12MPS)

**Fig. 2:**
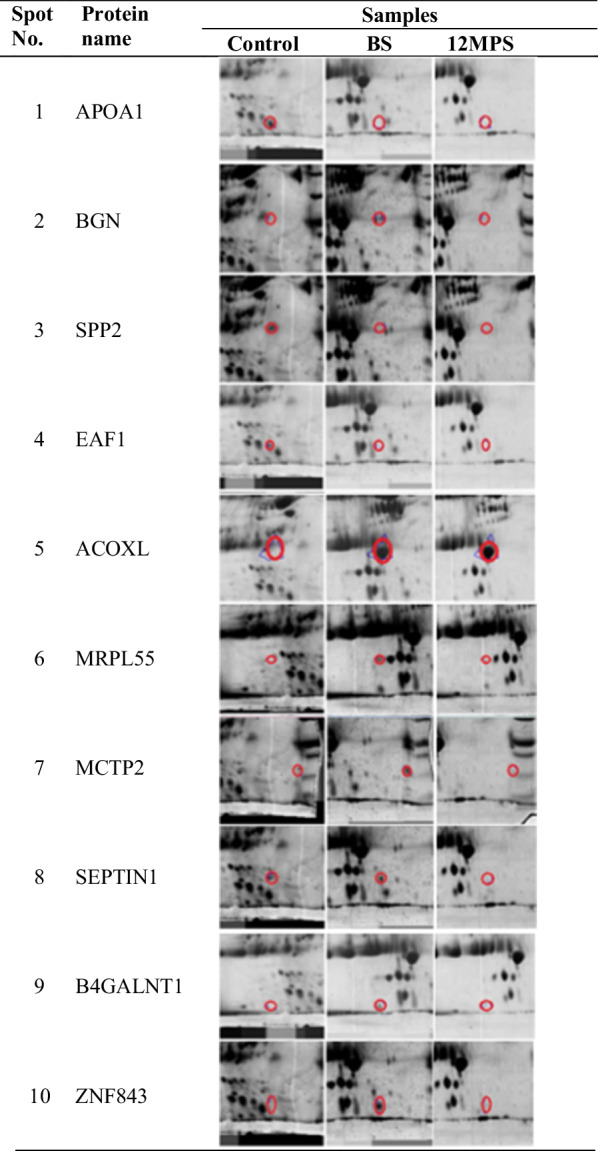
2DE gels stained with KANG method showing protein spots with different expression in the sera of BS, 12MPS patients and control groups. BS: Before Surgery, 12MPS: 12 Months Post-Surgery

A total of 10 protein spots were identified by mass spectrometry (LC–MS/MS) (Fig. 1). The expression change of each one in 2DE gel demonstrated in Fig. 2. Details about identified proteins are shown in Table 1.

**Table 1:** Details about each protein spot were identified by LC–MS/MS

Spot no	Protein name	Gene name	UniProt accession no	MW (Da)	pI	ANOVA	Fold	Control	BS	12MPS
1	Apolipoprotein A-I	APOA1	F8W696	10	8.03	2.617e−013	6.6	↑	↑	↓
2	Biglycan	BGN	C9JKG1	23	8.58	2.760e−011	2.1	↓	↑	↓
3	Secreted phosphoprotein 24	SPP2	Q13103	23	8.42	5.439e−012	2.7	↓	↑	↓
4	ELL-associated factor 1	EAF1	Q96JC9	12	7.22	8.027e−014	5.5	↑	↓	↓
5	Isoform 3 of Acyl-coenzyme A oxidase-like protein	ACOXL	Q9NUZ1-3	20	7.15	1.221e−015	2.5	↓	↑	↑
6	39S ribosomal protein L55, mitochondrial	MRPL55	A0A087X2A2	15	5.96	1.156e−011	3.5	↓	↑	↓
7	Multiple C2 and transmembrane domain-containing protein 2	MCTP2	F5H415	14	10	4.715e−010	2.6	↓	↑	↓
8	Septin-1	SEPTIN1	Q8WYJ6	15	7.82	2.454e−014	5.9	↓	↑	↓
9	Beta-1,4 N-acetylgalactosaminyltransferase 1	B4GALNT1	F8VR44	10	5.96	1.906e−012	4.8	↓	↑	↑
10	Zinc finger protein 843	ZNF843	ZN843_HUMAN	12	7.93	4.390e−010	3.7	↓	↑	↓

In the BS group, a total of nine spots with high expression proteins were identified as follows: Apolipoprotein A-I (APOA1), Biglycan (BGN), Secreted phosphoprotein 24 (SPP2), Isoform 3 of Acyl-coenzyme A oxidase-like protein (ACOXL), 39S ribosomal protein L55,mitochondrial (MRPL55), Multiple C2 and transmembrane domain-containing protein 2 (MCTP2), Septin-1 (SEPTIN1), Beta-1,4 N-acetylgalactosaminyltransferase 1 (B4GALNT1) and Zinc finger protein 843 (ZNF843).

On the other hand the expression of BGN, SPP2, MRPL505, MCTP2 and Septin-1 (SEPTIN1) has been decreased after surgery in 12MPS group. In control group APOA1 and ELL-associated factor 1 (EAF1) and ZNF843 were identified with high expression rather than in other groups.

**Fig. 3:**
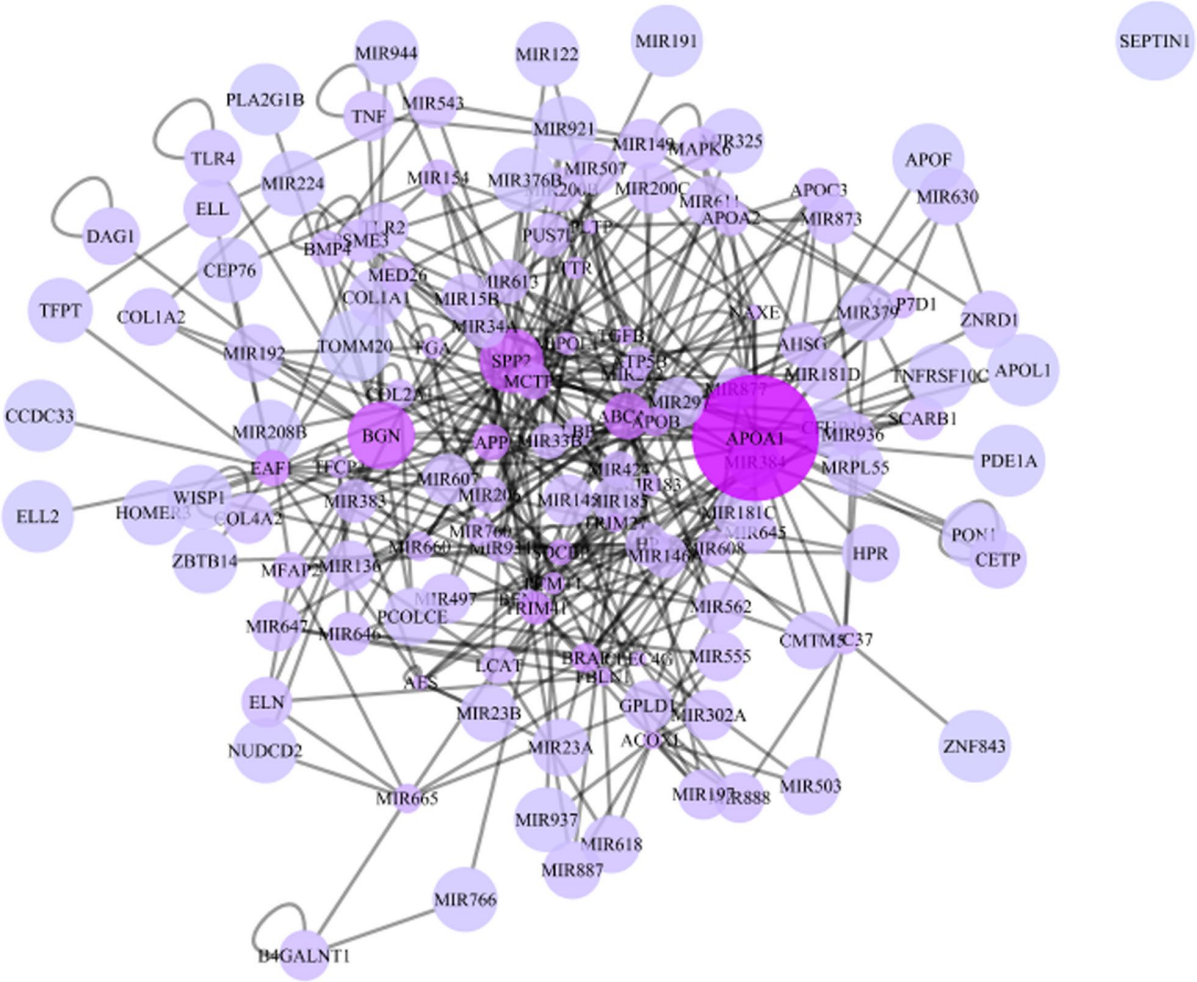
PPI network for lungs CE obtained from HPRD, BIND, MINT, INTACT and STRING databases by the application of Cytoscape v3.2.0

The whole network was visualized and analyzed using Cytoscape v3.2.0 software, including 137 nodes and 383 edges (Fig. 3).

The hubs, bottlenecks, and hub-bottlenecks are represented in the Tables 2 and 3. The topological parameters of network are described in Table 4. The finding indicates that 5 Hub-bottlenecks proteins including APOA1, BGN, SPP2, EAF1 and ACOXL are query proteins which were identified by MS analysis (Table 5).

**Table 2:** Bottlenecks proteins with significant centrality values, based on betweenness

No	Gen name	Protein name	Betweenness score
1	APOA1	Apolipoprotein A-I	5451.761
2	BGN	Biglycan	2832.578
3	SPP2	Secreted phosphoprotein 24	1978.989
4	APP	Amyloid-beta precursor protein	1656.169
5	ABCA1	Phospholipid-transporting ATPase	1425.333
6	EAF1	ELL-associated factor 1	1408.603
7	APOB	Apolipoprotein B-100	1124.131
8	MCTP2	Multiple C2 and transmembrane domain-containing protein 2	1046.588
9	TRIM41	E3 ubiquitin-protein ligase TRIM41	1002.482
10	BRAP	BRCA1-associated protein	651.1595
11	CDC37	Hsp90 co-chaperone Cdc37	503.4186
12	MIR760	–	501.196
13	SDCBP	Syntenin-1	495.0561
14	PCMT1	Protein-L-isoaspartate(D-aspartate) O-methyltransferase	463.2281
15	MIPOL1	Mirror-image polydactyly gene 1 protein	459.5198
16	CLEC4G	C-type lectin domain family 4 member G	453.8621
17	MIR665	–	422.4701
18	PLTP	Phospholipid transfer protein	421.2035
19	TGFB1	Transforming growth factor beta-1 proprotein	420.5315
20	ACOXL	Isoform 3 of Acyl-coenzyme A oxidase-like protein	415.5001

**Table 3:** Hub proteins with significant centrality values, based on degree

No	Hub genes	Protein name	Degree
1	APOA1	Apolipoprotein A-I	41
2	BGN	Biglycan	24
3	SPP2	Secreted phosphoprotein 24	23
4	ABCA1	Phospholipid-transporting ATPase	18
5	APP	Amyloid-beta precursor protein	15
6	EAF1	ELL-associated factor 1	15
7	MCTP2	Multiple C2 and transmembrane domain-containing protein 2	15
8	TRIM41	E3 ubiquitin-protein ligase TRIM41	14
9	APOB	Apolipoprotein B-100	13
10	BRAP	BRCA1-associated protein	13
11	SDCBP	Syntenin-1	12
12	PCMT1	Protein-L-isoaspartate(D-aspartate) O-methyltransferase	11
13	MIPOL1	Mirror-image polydactyly gene 1 protein	11
14	TGFB1	Transforming growth factor beta-1 proprotein	10
15	ACOXL	Isoform 3 of Acyl-coenzyme A oxidase-like protein	10
16	FBLN1	Fibulin-1	10
17	TRIM27	Zinc finger protein RFP	10
18	CLEC4G	C-type lectin domain family 4 member G	9
19	PLTP	Phospholipid transfer protein	9
20	NAXE	NAD(P)H-hydrate epimerase	9

**Table 4:** Topological features of PPI network

Topological parameter	Values
Number of nodes	137
Number of edges	383
Clustering coefficient	0.105
Network centralization	0.237
Network density	0.038
Network diameter	5
Average node degree	5

**Table 5:** Hub-bottlenecks proteins with highly degree and betweenness

Protein name	Hub degree	Bottleneck betweeness
APOA1	41	5451.761
BGN	24	2832.578
SPP2	23	1978.989
EAF1	15	1408.603
ACOXL	10	415.5001

## Discussion

The immunoproteomic approach to the diagnosis and follow-up the treatment outcome of human infectious diseases is an interesting tool [29]. Two-dimensional gel electrophoresis which is a main technique in the proteomics researches, has been one of the most effective methods for isolation of the proteins with complex components [33]. New proteomic tools allow high resolution analysis of complex proteins, through two-dimensional gels (2D-PAGE). The isolated proteins can be individually identified through mass spectrometry [34]. Proteomics investigation on sera components has been used to identify several biomarkers in infectious diseases including parasitic diseases [35–39].

The lack of evidence to support the use of serological tools for the follow-up of CE patients is an unsolved problem [19]. Keeping in mind the above limitations and due to the lack of markers and the relatively low serological efficiency, it is essential to finding new methods for monitoring the lungs CE in humans. Therefore, the development of new methods for follow up of the lungs CE especially in treated patients is essential [19]. Moreover, due to low sensitivity and incomplete specificity, the use of serology is restricted to case confirmation, especially among symptomatic patients with an atypical lung lesion. Serology may be of used as an indicator of relapse or recurrence [40–43]. Using new and applied methods in this regard is necessary [28]. Accordingly, mass spectrometry-based proteomics techniques, have arisen our ability to identify helminthes proteins [44].

Proteomic tools and bioinformatics analysis of sera from patients with evaluation of proteome levels related liver CE has been used successfully as a valid reliable diagnostic tool [45]. This method will increase the quality of the follow-up processes after a treatment [46].

Recently, proteomics has been used for liver CE follow-up on sera and plasma [47, 48]. So, using the above approach could be useful for evaluation of lungs CE after treatment.

In the present study, a novel approach was employed to identify candidate antigens for evaluation and following-up of human lungs CE after cyst removal. The use of 2D PAGE in combination with mass spectrometry, allowed us identification of ten different proteins using experimental conditions.

This approach has already been used a proteomics gel based method for sera evaluation of human liver CE patients [45]. Previous proteomics studies on hydatid fluid or larval stage of *Echinococcus granulosus* sensu lato (s.l.) have shown different proteins [49–51]. These identified proteins are involved in a wide range of biological functions including stress response, cytoskeleton, metabolism especially those which may participate in the mechanisms of cholesterol uptake from the host [52, 53]. Moreover, comparing their amino acid sequences has showed homology with related parasites [50].

Our results showed a total of 171 protein spots in all gels in the experiment. They were classified into three groups including: BS, 12MPS and control groups. Among them a total of ten protein spots have been selected for identification with mass spectrometry including: APOA1, BGN, SPP2, EAF1 ACOXL, MRPL55, MCTP2, SEPTIN1, B4GALNT1, and ZNF843. Some of these proteins have been reported earlier from hydatid fluid in sheep liver CE [54]. However, they have not been reported from human sera of CE patients, so far. Anyhow, based on centrality parameters of the PPI network (degree and betweenness) a total of five hub-bottlenecks proteins with significant centrality values including APOA1, BGN, SPP2, EAF1 and ACOXL were found. More analysis showed that APOA1 with high degree and betweenness as the first hub protein in the PPI network.

APOA1 is first important protein which was identified in patients’ sera with high expression before surgery. APOA1 is a potential ligand for monocyte and macrophage receptors [55]. These receptors may also be involved in plasma lipoprotein recognition and induce an anti-inflammatory phenotype in macrophages upon recognition of EgAgB. Apolipoprotein component, binds to monocytes and macrophages specifically, using receptors shared with plasma lipoproteins and seems to induce signaling events involved in the regulation of inflammatory pathways [55]. The antigenicity and immunogenicity of AgB has been reported to be associated with its protein moiety or apolipoprotein components [56]. Mass spectrometry used in our study showed the presence of APOA1 in sera of lungs CE patients. Due to usage of pooled sera samples in each group, cyst size or cyst location in the lungs are not applicable to our results. Interestingly, APOA1 has previously been detected in *Echinococcus granulosus* s.l. hydatid fluid [50, 55]. On the other hand, APOA1 has also been detected in *E. multilocularis* metacestode [57]. Apolipoprotein has been proposed to be involved in the uptake of lipids by *E. multilocularis* metacestodes more special in the uptake of cholesterol. In human cases this can interacts with human APOA1 a major constituent of cholesterol-transporting high-density lipoproteins in plasma [53]. Our findings showed that, this protein was present in the sera of lungs CE patients BS and was significantly decreased after removal of the cysts. So, it could be a good candidate to be introduced as an indicator of active lungs CE and could be used for follow-up and treatment programs of CE.

BGN is another protein which has been expressed at high level in sera of lungs CE patients. This protein has been decreased after lungs cyst removal in the patients in 12MPS group. This protein has been reported in *Trichinella spiralis* and has been proposed to contribute the control of inflammation [58]. In addition to necrotic and activated cells, the extracellular matrix can also release biglycan [59]. Serum BGN may be a new non-invasive indicative marker for the presence of non-alcoholic steato hepatitis (NASH) and liver fibrosis [60].

Serum biglycan might be used as a non-invasive marker of liver fibrosis and also can be a potential biomarker for lung cancer [61]. However, there was no data on BGN and echinococcosis. One of the important protein detected in our study was SPP2. The SPP2 is a protein which was identified in patients’ sera before surgery. After cyst removal the expression of SPP2 has been suppressed in 12MPS group. The reported data on SPP2, has shown that dramatically induces apoptosis of tumor cell. It has been shown that SPP2 dramatically inhibits cell proliferation [62]. However, its role in the CE is not known, yet.

A number of other proteins including EAF1 has been identified in lungs CE patients' sera. Also it was recognized as another hub protein in our PPI network. Comparison of EAF1 expression between control, BS and 12MPS groups showed the high level of EAF1 in control group. It seems that the cyst formation suppressed EAF1 expression. However, after cyst removal in 12MPS, EAF1 expression level in sera is still high.

ACOXL has not been detected in the control group while it has been detected in lungs CE patients' sera. It has been expressed at a high level, even 12MPS. It has not been detected in CE sera, so far. Our observation showed that the majority of pulmonary CE were adults and only one third of the patients were children; although, in general, pulmonary cystic echinococcosis infections are commonly seen in children [63, 64].

## Conclusion

The proteomics study revealed that in follow-up samples of lungs CE patients, the expression of several proteins was changed significantly 12 MPS groups in response to treatment and cyst removal. Such the five proteins as hub-bottleneck proteins were diagnosed. Among them APOA1 was more prominent. It can be concluded that change in expression of this protein in patients’ sera could be used as indicator tool for achievement of lungs CE surgical therapy.

## Data Availability

All data generated or analyzed during this study are included in this manuscript. The original datasets are available upon request to the corresponding author.
